# Assessing Alternative Pre-Treatment Methods to Promote Essential Oil Fixation into Cotton and Polyethylene Terephthalate Fiber: A Comparative Study

**DOI:** 10.3390/polym15061362

**Published:** 2023-03-09

**Authors:** Hanane Tansaoui, Nabil Bouazizi, Nemeshwaree Behary, Christine Campagne, Ahmida El-Achari, Julien Vieillard

**Affiliations:** 1 Laboratoire Genie et Materiaux Textile, University Lille, ENSAIT, 59000 Lille, France; 2 COBRA (UMR 6014), CNRS, INSA Rouen, UNIROUEN, Normandie Université, 55 rue Saint Germain, 27000 Evreux, France

**Keywords:** geranium essential oil, textile fabrics, treatment methods, antibacterial activity, eco-friendly, potential applications

## Abstract

This study aims to develop a new refreshing feeling, ecological, and antimicrobial fabrics for medicinal applications. The geranium essential oils (GEO) are incorporated into polyester and cotton fabrics by different methods, such as ultrasound, diffusion, and padding. The effect of solvent, nature of fibers, and treatment processes were evaluated via the thermal properties, the color strength, the odor intensity, the wash fastness, and the antibacterial activities of the fabrics. It was found that the ultrasound method was the most efficient process for incorporation of GEO. Ultrasound produced a great effect on the color strength of the treated fabrics, suggesting the absorption of geranium oil in fiber surface. The color strength (K/S) increased from 0.22 for the original fabric to 0.91 for the modified counterpart. In addition, the treated fibers showed appreciable antibacterial capacity against Gram-positive (*Staphylococcus epidermidis*) and Gram-negative (*Escherichia coli*) bacteria strains. Moreover, the ultrasound process can effectively guarantee the stability of geranium oil in fabrics without decreasing the significant odor intensity and antibacterial character. Based on the interesting properties like ecofriendliness, reusability, antibacterial, and a refreshing feeling, it was suggested that textile impregnated with geranium essential oil might be used as a potential material in cosmetic applications.

## 1. Introduction

Biofunctional textiles are materials that exert a biological impact on human skin. Many textiles constitute the basis for the delivery system of cosmetic or pharmaceutical substances when the textiles are exposed to the skin [1]. Bioactive substances are commonly incorporated in textiles for their antibacterial, antifungal, and anti-inflammatory activities. The substance may release under specific conditions to be absorbed by the skin. These cosmetic and medicinal textiles, with a slow release of the active compound into the skin, can be useful for people with sensitive skin. In this regard, there are numerous commercialized textile products currently on the market that claim to have characters usually found in cosmetics [2], such as moisturizing, slimming, energizing, antibacterial, fragrance, refreshing, as well as UV protection [3,4]. Therefore, textile treatments are still under development to obtain performant and durable fabric [5]. In the cosmetic area, natural and eco-friendly products like biopolymers and essential oils (EO) are mainly desiredto obtain antibacterial and refreshing textiles.

Essential oils (EO) are mixtures of volatile and semi-volatile materials obtained from natural sources by distillation, or by some other suitable cold pressing methods [6,7]. Rose-scented geranium is a well-known fragrant plant belonging to the medicinal family of *Geraniaceae* [8]. Geranium essential oil (GEO) is obtained from the leaves of geranium and is considered not only the base for the perfume and cosmetic synthesis, but also for food industries. In addition, GEO has been used for a long time for the treatment of diarrhea, diabetes, inflammation, and gastric ulcers [9,10]. It has exhibited a variety of medicinal properties such as antimicrobial, immunostimulant, antioxidant, hypoglycemic, and anti-inflammatory characters [11,12,13].

As mentioned above, clothes are considered an important product in people’s lifestyles. In this case, textile materials with a refreshing feeling and antibacterial qualities are of interest. Recently, there is an increased interest in using natural products in textiles because of their biological activity, biocompatibility, and low toxicity. There is a growing demand in the market for biologically based textiles with functional properties such as antimicrobial and a refreshing feeling [14]. Nabil et al. studied the surface modification and functionalization of cellulosic fabrics using atmospheric non-thermal plasma treatment technology as an eco-friendly and promising alternative substrate for a full application, particularly the antimicrobial activities [15,16]. As is known, the biologic finishing on textiles was applied to serve a vital role in the human body, and it can be considered a medicinal product [14]. The most convenient and efficient application of textile fabrics for incorporation and transporting bioactive compounds to the skin was to load the textile with plant extract [17].

Nowadays, there are various technical methods for preparing refreshing and biomedical textiles to improve their efficiency and stability, control their releases of its active compound, and ease their application. For example, Nabil et al. demonstrated that incorporation of bioactive neem oil over cellulosic fiber using an environmentally sound approach induces a high antibacterial and antiradiation characters for the resulting textile material [18,19].

Various synthetic compounds have been applied to fabric to impart antibacterial activity with good durability. However, most of them are toxic to humans and are not biodegradable. The main concerns related to synthetic antimicrobial textiles are the increase in bacterial resistance to the biocides, the possibility of triggering allergies and side effects in the users, and water pollution [20]. In addition, various herbal products with active antimicrobial ingredients, such as aloe vera, tea tree oil, eucalyptus oil, prickly chaff flower and tulsi leaf, clove oil, and many more, have also been used for this purpose.

Encapsulated citronella EO on cotton fabric was used to enhance the repellent retention time with 90% activity [21]. Lemon eucalyptus EO with 85% citronellal and silica–lavender was fit into textile material to achieve mosquito repellent properties along with a pleasant fresh scent [22]. Despite the distinctive properties produced by the essential oil, reusability and sustainability are strongly needed. Herein, the chemical fixation of EO is insufficient to achieve good reusability, and the properties of these oils can be released rapidly. Therefore, effective methods are required for ecological and sustainable fixations of EO into textile.

In this direction, the aim of this study is to incorporate GEO via various treatments methods such as ultrasound, diffusion and padding process.

In this work, we investigated the effects of the solvent and the type of treatment on the final properties of the fabrics. The olfactory tests and color strength measurements of cotton and polyester fabrics are reported here. To the best of our knowledge, no published literature reported the effects of treatments method on the fixation of GEO in fabrics. The obtained materials were characterized and the results are discussed. Moreover, the antibacterial and the sustainability characteristics of the prepared fabrics were investigated.

## 2. Materials and Methods

### 2.1. Materials

Geranium essential oils (GEO), petroleum ether, and ethanol (C_2_H_5_OH) were purchased from Sigma Aldrich (St. Louis, MI, USA). Deionized water used in all experiments was obtained using a water purification system provided by GFL mbh Ltd (Berlin, Germany).

A 100% polyester–polyethylene terephthalate (PET) and 100% cotton (CO) fabrics were purchased from Subrenat (Ref. UB1-FROM/CR1.2) (Mouvaux, France).

#### 2.1.1. Air Atmospheric Plasma Treatment of PET Fabrics

In order to remove impurities and spinning oil present on the surface of nonwoven surface, the PET fabric was cleaned according to the methods described in our previous work [23].

**Scheme 1:**
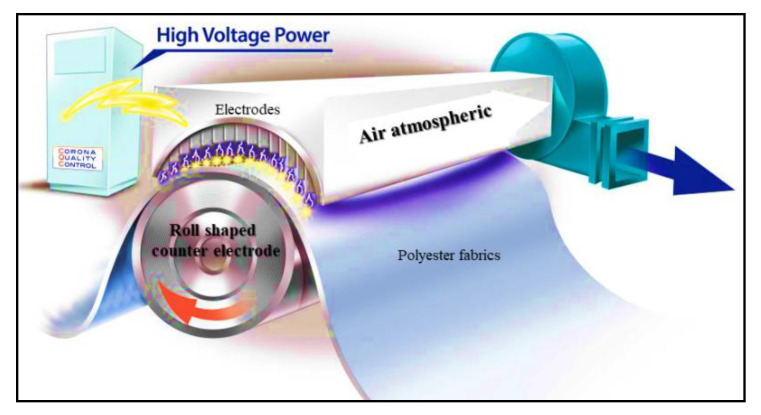
Schematic flow diagram of air atmospheric plasma treatment.

(i) First, PET fabric was cut and engrossed into the petroleum ether solution using Soxhlet for 5 h at 60 °C;
(ii) Second, nonwovens were ultrasonically impregnated in absolute ethanol for 20 min followed by drying for 12 h. In this step, nonwovens were repeatedly ultrasonically washed with deionized water and dried three times;
(iii) Third, the cleaned fabrics were activated by atmospheric air plasma, based on a dielectric barrier discharge (DBD), which was used to activate the PET surface. The treatment was carried out by the ”Coating Star” plasma treatment set-up provided by Ahlbrandt System (Lauterbach, Germany) as shown in Scheme 1. Atmospheric air was chosen as the gas, and the following parameters were used to treat the nonwoven on both sides: a frequency of 26 kHz, rotation speed of 2 m min^−1^, electrical power of 750 W, an inter-electrode distance of 1.5 mm, and a plasma treatment power of 60 kJ/m^2^. The following machine parameters, as shown in Table S1, were kept constant throughout the treatments of all samples. The plasma treated PET was denoted P-PET.

#### 2.1.2. Ultrasound-Assisted Oil Absorption into Fabrics

Ultrasound-assisted oil fixation on fabric surface was employed to ensure high adhesion on the surface of the material and good absorption of essential oil molecules into the fibers, without affecting their properties. During the process, 40 mL of methanol, 1 g of essential oil, and 2 g of the samples were added to a flask at room temperature (Table 1). The mixture was placed in an ultrasonic bath (KQ 500DV, Kunshan Ultrasonic instruments Co., Ltd., Kunshan, China) with a power of 200 W and a frequency of 40 kHz. The temperature of the bath was set at 30 ± 1 °C during 30 min. Then, the mixture was separated by simple filtration, and the obtained samples were dried at 50 °C overnight. The final products were denoted P—PET—G for the polyester fabric modified by GEO, and CO—G for the GEO modified cotton fabric.

#### 2.1.3. Padding Method for Essential Oil Incorporation

A laboratory-scaled padder (Werner Mathis AG, Switzerland) was used to incorporate GEO into the PET and CO fabrics by the padding process. The pressure was fixed to 2 bars, and the rotation speed was fixed to 2 m/min. A solution containing 1 g of GEO and 40 mL of methanol was prepared initially, and then placed between the rollers, at room temperature (Table 1). The fabrics were padded during 10 min, and around five cycles were needed to achieve the complete impregnation of fabrics and good fixation of GEO. After that, the treated samples are dried at 50 °C for 12 h.

#### 2.1.4. Diffusion Method for Essential Oil Insertion

Diffusion method was used for insertion of GEO into the PET and CO fabrics. Typically, a solution of 10 mL ethanol, 140 mL distilled water and 2% wt GEO were stirred in metallic bottle for 1 h (Table 1). Then, cotton and polyester fabrics were added to the mixture solution and kept for heating, during 4 h at 90 °C for cotton and 130 °C for polyester fabric. After that, the treated samples are dried at 50 °C for 12 h.

### 2.2. Surface Analysis

Surface morphologies of all fabrics were analyzed by scanning electron microscopy (JEOL JSM IT100, JEOL Ltd., Tokyo, Japan). Thermogravimetric analysis was performed by a thermogravimetric analyzer (TG-DTA, A6300R) with a heating rate of 10 °C/min from room condition to 500 °C along with differential scanning calorimetry (DSC). To quantify the essential oil releases from the fabrics, the residue solution obtained after washing was analyzed by a UV-VIS spectrophotometer in the 200–600 nm range. In order to quantify the degree of hydrophilization of the prepared cotton and polyester surface, wettability measurements were carried out before and after fixation of the essential oil. The water contact angle of all fabrics was measured by tensiometer and digidrop analysis.

### 2.3. Color Strength

The measurements of color strength of CO and PET fabrics treated with GEO were carried out according to the procedure published previously [24]. The reflectance values of the treated samples were measured at different wavelengths using a Datacolor spectrophotometer (Model: DC 550, Datacolor International, Rotkreuz, Switzerland) interfaced to a personal computer and the color strength was obtained via the following equation:

$$\frac{K}{S}=\frac{\left(1-R\right)2}{2R}$$

where *K* is the absorption coefficient, *S* is the scattering coefficient, and *R* is reflectance value.

### 2.4. Solubility Parameters of the Geraniol Essential Oil and Polyester

The Hildebrand solubility parameter named δ_t_ was obtained from Hansen Solubility Parameters (HSP). According to Charles Hansen, the mains feature of solubility is the result of the contribution of three types of interactions: dispersion forces (δ_d_), polar interactions (δ_p_), and hydrogen bonds (δ_h_) [25].

The HSP values of polyester polymers were obtained from the web-based solubility parameter data base which considers the HSP values [26] and that of the geraniol from a past published paper [27]. The Hildebrand solubility parameter δ_t_ was obtained via Equation (1)

δ^2^_t_ = δ^2^_d_ + δ^2^_p +_ δ^2^_h_ (1)

Table 1 summarizes more detailed values of both HSP and the Hildebrand solubility parameters. The Hildebrand solubility calculated from HSP for PET is given in Table 2 (δ_t_ = 21.4 MPa^1/2^) and is in good agreement with those obtained by Slark; that is, δ = 21.9 MPa^1/2^ at 129.4 °C for polyester fiber-based fabrics [28].

### 2.5. Testing of Olfactory Function

In order to investigate the prepared samples, the odor intensity was evaluated on the basis of olfactory testing, where 10 investigators are asked to score between 0 (none) to 10 (excellent) rate choices (Table S2). Its validity was established in large groups of investigators. Although it is understood that such brief tests of olfactory function cannot identify each person with olfactory loss simply due to the small number. The olfactory test consisted of fabrics odorants, which were selected based on the geranium fragrance identification and pleasant olfactory character.

### 2.6. Durability and Rub Fastness Test

In order to study effect of wash cycles on the treated samples, a number of three successive washes were carried out under the conditions of washing with a domestic machine with Mathis Labomat equipment. The rubbing test was carried out on a martindale, according to the following protocols. According to NF EN ISO 12947 standard, samples are subjected for 1000 rubs on the martindale for three successive washes with purified water, at 30 °C, for 30 min with air dry between each wash. Also, the olfactory test after each washing cycle was used to investigate fabric reusability and stability.

### 2.7. Antibacterial Tests

The antibacterial activity of prepared cotton and polyester fabrics were evaluated by means of disc diffusion and zone inhibitory for two types of bacteria, as Gram-positive *Staphylococcus epidermidis* (ATCC 12228) and Gram-negative *Escherichia coli* (ATCC 25922) bacteria strains according to the method explained elsewhere [29]. The antibacterial behavior of the samples was obtained on individual bacteria. The test is a semi-quantitative method where the antibacterial activity was assessed by examining the absence or presence of microbial growth in the contact zone between agar and specimen and on the eventual appearance of an inhibition zone according to ISO 20645. The textile sample with 1 cm^2^ were placed on the surface of nutrient agar medium, which was swabbed with the bacterial (10^6^ cfu/cm^3^) culture. The plates were incubated at 37 °C for 24 h to measure the zone of inhibition in millimeters formed around the textile. The results are expressed as the width of the inhibition zone (mm).

## 3. Results

### 3.1. Effects of Solvent

**Scheme 2:**
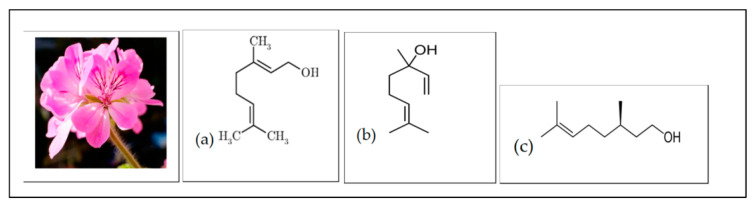
Chemical structure of the geranium essential oil: (**a**) geraniol, (**b**) linalool, and (**c**) citronellol.

**Figure 1:**
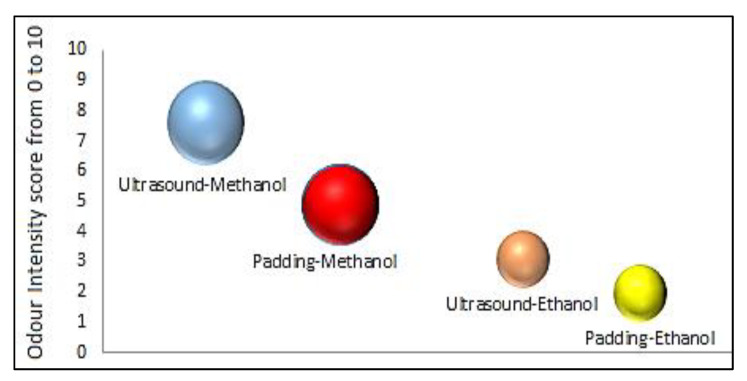
Effect of methanol and ethanol on the odor intensity for ultrasound and padding methods for PET and CO.

In order to identify the significant factors considered in the present study, the effects of temperature, time, and solvent were studied. The effect of solvents on the absorption of EO was examined by impregnation of cotton fabrics in two different solvents, such as methanol and ethanol, with the addition of fixed geranium oil under the same conditions. Figure 1 shows that methanol is more suitable for the incorporation of geranium oil, as compared to the ethanol. Accordingly, the results were the same for each method of preparation, that is, ultrasound and padding, suggesting that methanol played a significant role on the EO impregnation. In contrast, ethanol displayed the lowest results for both methods, indicating that ethanol solvent may affect the absorption of EO into fabrics. These results were confirmed by olfactory tests (discussed in the following sections), which indicated that methanol results in high odor intensity of EO in fabrics (Table 1). Consequently, methanol can be considered a good candidate for EO absorption. In this regard, an explanation can be postulated in terms of interaction and the affinity of methanol with essential oils. As is known, geranium oil contains geraniol molecules which can be easily interact with a polar solvent. Hence, the geraniol involved interaction with methanol with the liberation of water [30]. By comparing the results between ethanol and methanol solvents, the difference could come from to the number of the Van der Waals force, where methanol has weak intensity and then strong affinity to interact with external molecules like geraniol (Scheme 2). Further increasing the concentration of methanol leads to the formation of narrower holes resulting in smoothing of the fiber surface. This behavior can be described as below. The interaction of geranium-oil–methanol is strong, since methanol is a good solvent. Therefore, the etching process becomes uncontrollable and causes breaking of the fiber. A mixture of methanol and geranium oil should be miscible as both are polar and can interact with each other using hydrogen bonds. Thus, the hydrogen of hydroxyl groups, present in solution, readily bonds with the oxygen in ester groups present on fabrics. Subsequently, the methanol encourages the penetration of EO into the fiber surface by hydrogen bonds leading in the formation of different surface smoothing and roughness, as supported by the SEM analysis (discussed in the following sections). Consequently, using pure solvent like methanol provides a strong and deep penetration of EO into polyester and cotton fibers [31].

### 3.2. Morphological Analysis

**Figure 2:**
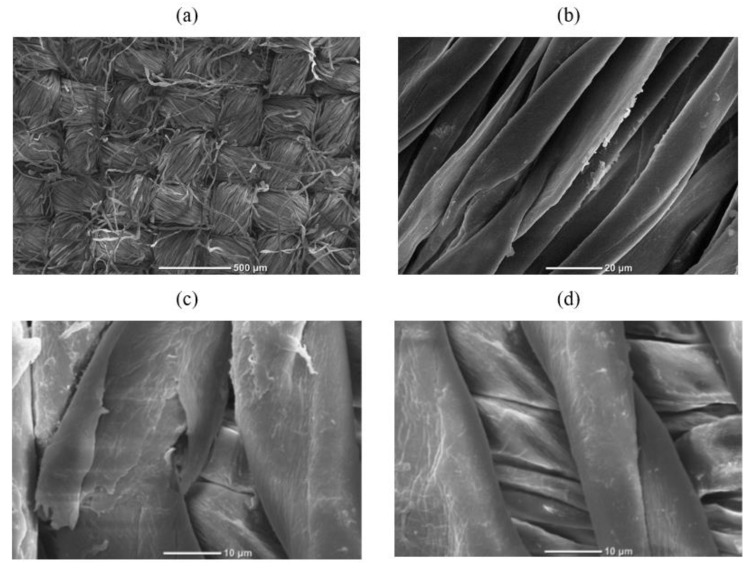
SEM images of (**a**) CO and (**b**–**d**) CO—G: Visualization of the geranium oil distribution on cotton fabrics.

**Figure 3:**
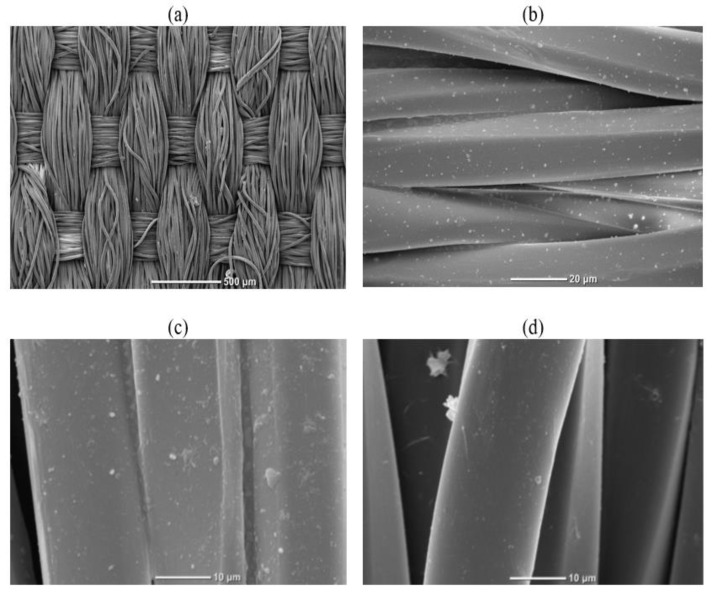
SEM images of P-PET (**a**) and P—PET—G (**b**–**d**): Visualization of the oil distribution on polyester fabrics.

**Figure 4:**
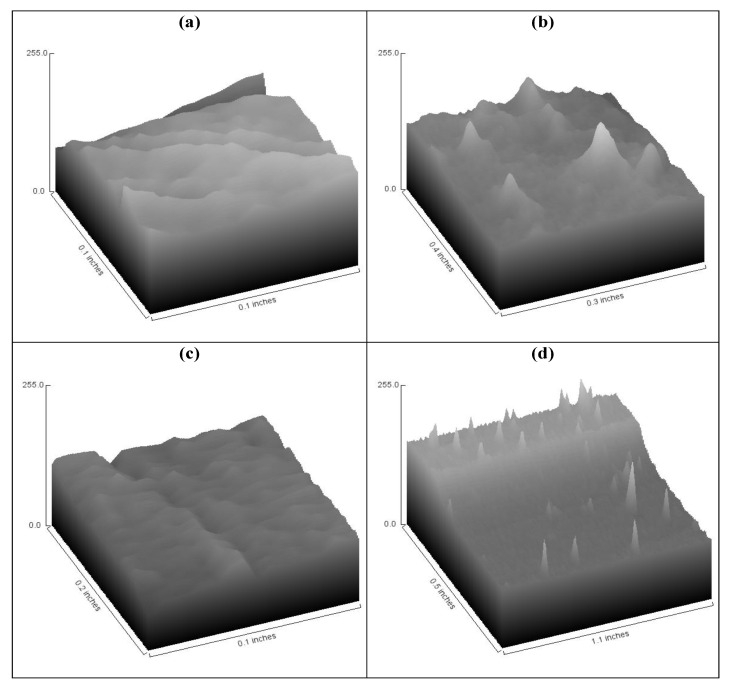
Surface morphological plot of CO (**a**), Co—G (**b**), P—PET (**c**), and P—PET—G (**d**).

In order to confirm the successful fixation of GEO, surface morphology of unmodified and modified samples were examined by scanning electron microscopy (Figure 2 and Figure 3). As seen, the fibers surface of both the starting materials PET and CO fabrics were intact and smooth before the fixation of GEO. However, after the oil fixation, visible changes were observed indicating the formation of a film layer at the surface of the fibers (Figure S1). It was clear that GEO induced the exfoliation of the fiber, suggesting the absorption of essential oil into the fibers. Furthermore, GEO fixation caused a greater number of pores with smaller size than the untreated fabrics. This can be explained by the entire fixation of essential oil into the fabrics surface. Figure 4 corresponds to the surface morphology determined using Image-J software, which visualized the smoothing and roughness properties of the fiber surface. Clear differences are shown between untreated and treated fabrics. GEO incorporating CO and P-PET displayed a roughness surface, whereas the original counterparts present a smooth surface. Importantly, the surface roughness was clearly improved due to the EO absorption in fibers. Moreover, incorporation of GEO, as an active agent decreased the surface energy of the fiber fabrics [32]. Accordingly, the roughness and chemical composition of CEO were a key factor of the slight increases in the hydrophilicity, as supported by the wettability measurements (discussed in the following section). Afterwards, surface roughness was reinforced by geranium oil absorption, and its low surface energy was caused by ultrasound vibration.

### 3.3. IR Evidences for GEO Fixation

**Figure 5:**
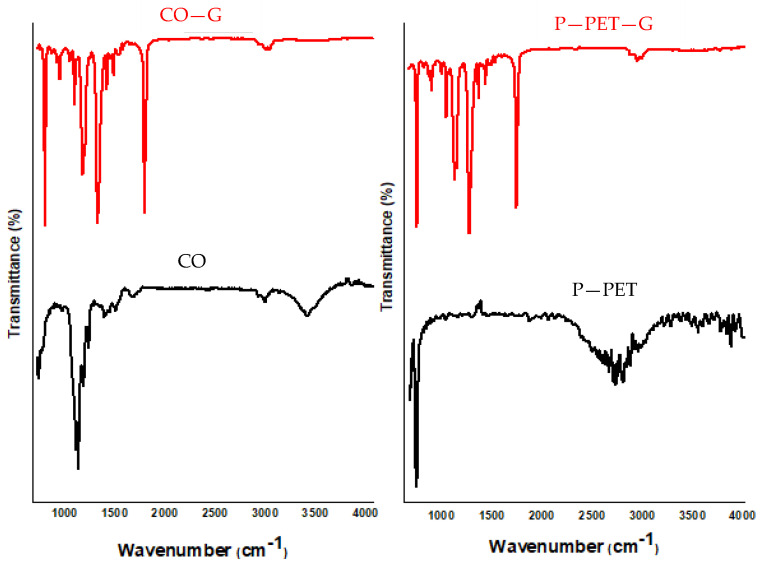
IR spectra of P—PET, CO, P—PET—G, and CO—GEO materials.

Infrared studies were carried out to approve the fixation of GEO at the PET and Co surfaces (Figure 5). The IR spectra of original PET, displayed some bands in the region 1720–650 cm^−1^, which were attributed to the stretching vibration of CH_2_, C=O, and aromatic C=C, indicating the structure of polyester fabric. While the IR spectra of Co showed more peaks between 600 and 1700 cm^−1^, indicating the structure of cotton as confirmed by our previously published work [1]. Focusing on the IR spectra of Co—G and P—PET—G materials, it’s clear that several functional groups are added after GEO fixation, as explained by the visible changes between the spectra. A confirmation of the GEO molecules at the Co and PET surface are evidenced by the strong peak at 3350 cm^−1^, due to the stretch vibrations of (O–H) bonds of the GEO. In addition, the wide peaks formed at 2960 and 2870 cm^−1^ are associated with the asymmetric stretching vibration of CH_2_ and CH_3_ of the methyl group over GEO [2]. Another confirmation of GEO grafting at Co and PET surfaces are obtained by the bond at 2728 cm^−1^ related to C=O vibration. Accordingly, the GEO molecules were successfully fixed at the surface of PET and Co materials.

### 3.4. Hydrophilic Character and Thermal Stability

**Figure 6:**
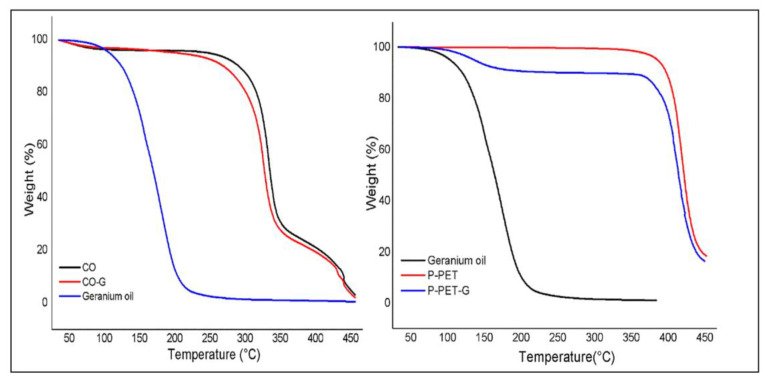
TGA curves of geranium oil, cotton, polyester fabrics, and their modified counterparts.

Contact angle measurements of prepared samples, under ambient conditions are summarized in Table 3. As a first overview, results demonstrate a limited influence of geranium on cotton as compared to PET. The hydrophilic character increased on P—PET—G (73°) as compared to the starting P—PET (89°). Particularly for PET fabrics, this accounts for a rise of the hydrophilic surface associated the appearance of an OH stretching bond. Such interactions impose diffusion hindrance that causes wettability without affecting the hydrophilic character. A confirmation in this regard was obtained through the low weight loss of 2–5% of CO—G and P—PET—G in the temperature range of 40–160 °C, as measured by TGA analysis in air at a 20 °C·min^−1^ heating rate (Figure 6). 

This weight loss mainly accounts for dehydration and most methanol volatilization, and is paradoxically in the same magnitude order as compared to the other samples. A much higher weight loss of ca. 95–98% occurred in the range 200–350 °C, and that was attributed to diverse processes according to the sample (Table 4). As expected, geranium EO exhibited slightly lower thermal stability up to ca. 200 °C as compared to CO—G and P—PET—G, which represent higher thermal stability up to 350 °C. Their almost similar thermal stability must mainly be due to that of their common hydroxyl incorporated by geranium oil absorption in the fiber surface. Consequently, TGA measurements confirmed the successful immobilization of geranium oil into polyester and cotton fabrics.

Moreover, as observed in the thermogram profiles in Figure 6, the essential oil of geranium is decomposed at approximately 200 °C, presenting a single thermal event of decomposition and without residual percentage. However, concerning CO—G, two distinct regions of mass loss were observed. This fact has happened due to the absorption of geranium EO into fabric surface, which came to occupy the place previously taken by the molecules of water, as described by Venturini et al. [33,34]. With the increase in temperature, the geranium EO is released with the decomposition of the biopolymer that protects it. Consequently, the thermal stability of the geranium EO modified fabrics was improved. This stability would be sufficient for the durability of treated fabrics. Furthermore, TGA analyses is generally taken as a proof of real inclusion. In addition, the increment of thermal stability may be propitious to the processing and storage of GEO.

### 3.5. Olfactory Properties of Treated Fabric

**Figure 7:**
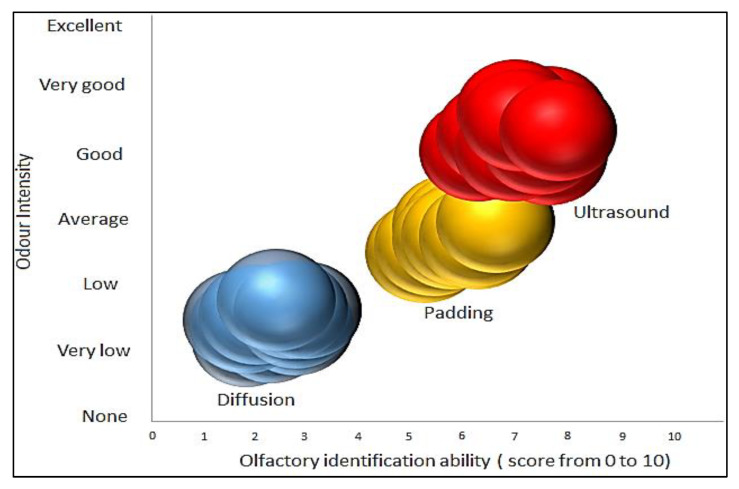
Comparison of olfactory stimulation effects on the treatment methods.

To assess whether the geranium oil differences could be incorporated with changes in the treatment methods, all prepared samples were subjected to the olfactory behavioral test. We stimulated the olfactory identification ability with odor intensity as well as several scores from 0 to 10 used in the behavioral test. The olfactory properties in odor intensity obtained for treated polyester and cotton fabrics is shown in Figure 7, where the relationship between the olfactory score and each sample as function of methods were evaluated. A first overview was obtained on the successful incorporation of geranium oil into both fabrics, as supported by the positive score rates, which ranged from low to near excellent odor intensity. Results demonstrated that investigators had significantly increased olfactory identification abilities of samples obtained from an ultrasound as compared to the other samples. Therefore, an ultrasound is the performant process for EO absorption, particularly geranium oil as supported by the rate score around 9 with very good odor intensity. By comparing the results between the three methods, the diffusion process appears to be enable to the penetration facility of EO into fabric, caused not only by the weak stability of EO during the preparation, but also the high heterogeneity of solvent–essential-oil that decreases the absorption of EO in fabrics. Unlike the insufficient results obtained from the padding method, EO can be penetrated with appreciable quantity into fabrics, indicating that the padding process immobilizes the geranium oil via weak adsorption at the fiber surface. In this regard, diffusion and padding methods present negative effects for the absorption of EO, which causes the oil loss in term of odor intensity.

While near excellent odor intensity for fabric is obtained from the ultrasound method, it is possible that the essential oils drops readily impinge on the host surface, and then improve the retention of geranium in cotton or polyester fabrics and consequently increase the durability. The above results are in good agreement with those obtained from TGA.

### 3.6. Color Strength Evidence of EO Absorption into Fabrics

**Figure 8:**
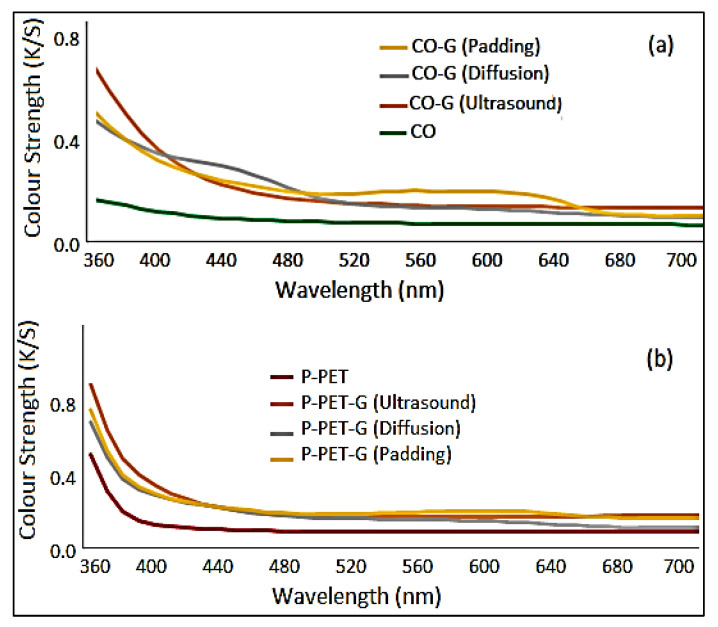
Effect of treatment methods on the color strength of cotton (**a**) and PET (**b**) fabrics treated with geranium essential oil.

In this study, the measurements of color strength were employed to show not only the successful absorption of geranium oil into polyester and cotton fabrics, but also to compare and evaluate the treatment methods [35]. In order to deduce the correct conclusion, the measurement was carried out under normal condition for all samples. Effects of ultrasound, diffusion, and padding treatments on the color strength of fabrics are shown in Figure 8. It is shown that the color strength of both fabrics was higher for the data of the ultrasound, which present 0.91 vs. 0.81 and 0.77 on the P—PET—G and 0.79 vs. 0.56 and 0.50 on the CO—G treated via padding and diffusion methods, respectively. The highest color strength (0.91) was shown in the fabric treated by ultrasound, whereas the lowest color strength (0.50) was obtained by the fabric treated via the diffusion process. Consequently, it can be assumed that GEO were absorbed into the polyester and cotton fiber via the used treatments process, particularly the ultrasound which significantly showed the incorporation of GEO. The achieved results are consistent with those obtained by SEM, TGA, and olfactory tests. The higher color strength of the treated fabrics is caused by the interaction between the polar functional groups on the geranium oil and the hydrophilic nature of the fiber to allow differences in the surface color. Among the treated samples, only P—PET—G and CO—G fabrics obtained from the ultrasound method gave the highest K/S values. Again, this trend was contributed by the combined effect between the GEO absorption and roughness surface.

### 3.7. Durability/Fastness Properties

**Figure 9:**
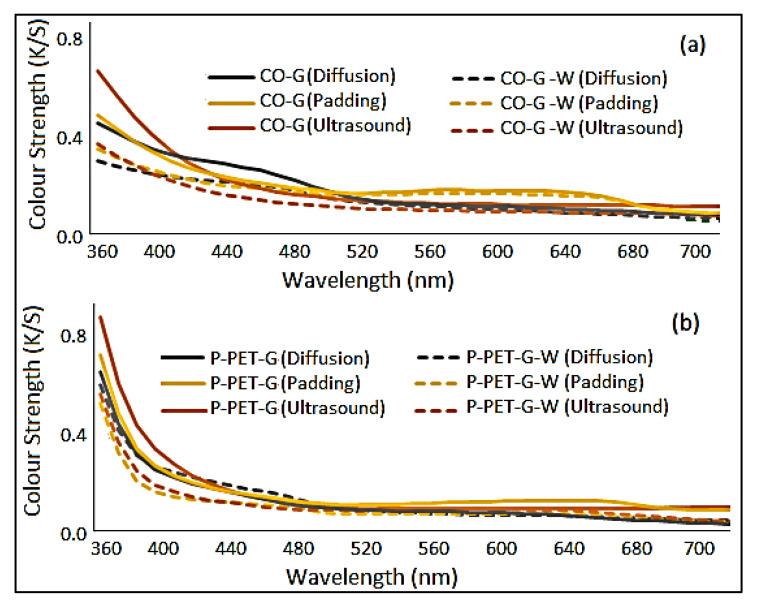
Effect of washing cycles on the color strength of cotton (**a**) and polyester (**b**) fabrics treated with geranium essential oil by different treatments.

In order to evaluate the durability of the prepared samples, ultrasound, padding, and diffusion methods-assisted essential oils incorporating on CO and P-PET fabrics were equally subjected to washing cycles (Figure 9). At the end of washing process, the samples were rinsed in soft water and left to dry under ambient conditions. As a result, it is clear that sample from the ultrasound assisted-essential oil incorporation yields the highest K/S, as compared to the other treated samples. However, the wash fastness was low and indeed the first wash removed surplus physical desorbed GEO. The essential oils showed acceptable dry and wet rub fastness after the first wash for cotton fabric, whereas this was not the case for the PET sample.

Interestingly, results from the ultrasound process demonstrates that even after several washing cycles, GEO persisted in the both polyester and cotton fabrics. These results are consistent with those obtained for odor evaluation without scratching the fabrics. This was expected since the low hydrophilicity character is an olfactory effect mainly dependent on the quick evaporation of the active products. Therefore, any increase in the oil content of treated textiles would represent an increase in the durability.

However, the odor intensity of all samples decreased after the washing process, caused by the volatilization of geranium oil during the storage time.

No significant decreases in odor intensity were observed among the samples from ultrasound methods, confirming again the efficiency of this technique in comparison to the other methods.

The slight reduction of odor intensity results indicate that washing did not greatly affect the stability in terms of geranium oil releases. In this regard, a confirmation was established from the wash fastness process that indicates the persistence of GEO odor after several washing cycles (Figures S2–S6). Despite fewer washing cycles as compared to the above results, it is recommended to introduce ultrasound technologies to assist the EO fixation.

### 3.8. Bacterial Susceptibility Testing to Antibiotics

**Figure 10:**
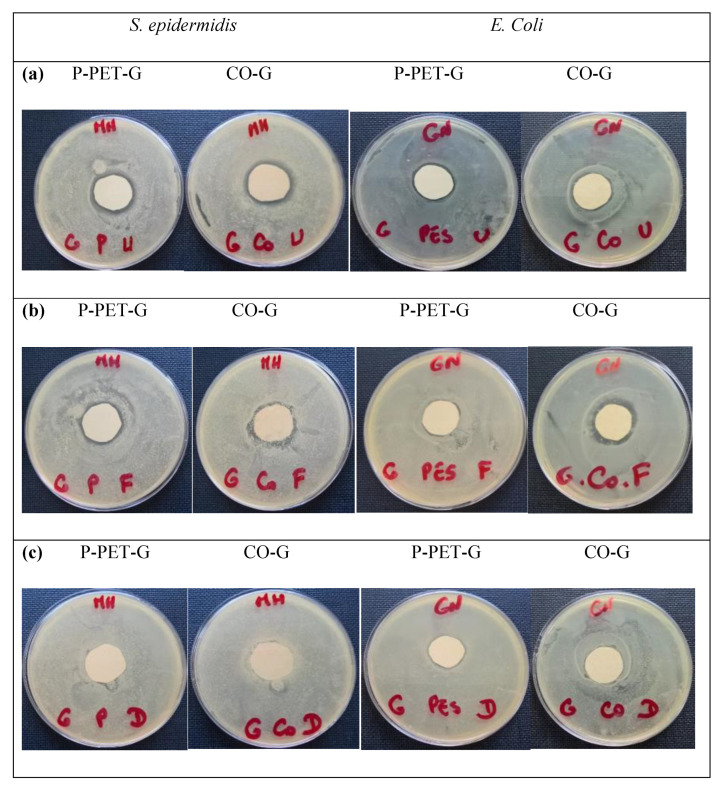
Inhibition zones revealing the antibacterial activity at 37 °C of CO—G and P—PET—G prepared via (**a**) ultrasound, (**b**) padding, and (**c**) diffusion methods.

In this study, the antibacterial activity of the geranium treated fabrics is evaluated for two bacteria (*E. coli* and *S. epidermidis*), and the results are shown in Figure 10. The results show no response for the untreated samples, where the bacteria remain over the surface of fabric, suggesting the absence of resistance to bacteria. However, after incorporation of GEO, all samples showed affinity towered the inhibition of both bacteria. The results were obtained from inhibition zone that surrounded the fabric surface. This can be explained by the key role of geranium oil absorption that introduce a visible difference on the antibacterial activity for each sample. By comparing the inhibition zone between samples prepared through the ultrasound, diffusion, and padding methods, it is clear that ultrasound caused an obvious inhibition zone against both bacteria for polyester and cotton fabrics. Results can be explained by the key roles of the ultrasound process on the incorporation of GEO, and consequently improving the antibacterial activity. The visible changes on the inhibition zone revealed the antibacterial property of GEO; in particular, the results associated with the ultrasound as compared to the other methods. As seen, the Gram-positive bacteria *S. epidermidis* was less affected by geranium-treated fabrics than the Gram-negative bacteria *E. coli*. Here, the nature of the cell wall structure was one of possible reasons for the sensitivity. *S. epidermidis* is composed of multilayers of peptidoglycan with an abundant number of pores that renders them more susceptible to reactive species, leading to the cell damage. However, the cell wall of *E. coli* is relatively thin which would be less vulnerable to the attack of reactive species. In this regard, geranium oil contains mainly geraniol responsible for the inhibition of both bacteria. Therefore, treated fabrics displayed better antibacterial activity against *E. coli* than *S. epidermidis*. Interestingly, the resistance of the geranium-treated fabrics against washing is one of the major concerns of antibacterial fabrics [36,37]. To investigate whether the coated fabrics would retain its antibacterial property after subsequent washing cycles or not, the prepared fabric was subjected to successive washing cycles, and the antibacterial rates were measured after three washing cycles. As can be seen in Figure S6, both polyester and cotton samples retained their antibacterial properties after three washing cycles. In fact, the inhibition rate for *S. epidermidis* and *E. coli* were slightly decreased. This result indicates the strong fixation of geranium oil to the surface of fabrics via the ultrasound method. From the above discussions it can be concluded that the ultrasound-assisted geranium absorption actively inhibits the growth of bacterial pathogens.

The diameter of the inhibition zone against *E. coli* and *S. epidermidis* (Table 5) was found to vary according to the treatment method and bacteria. Based on the diameter of the inhibition zone value, it clearly appears that the sample obtained from the ultrasound method showed an effective resistance against bacteria. Ultrasound induced the strongest diffusion rate and antibacterial activity with higher efficiency against *E. coli* than *S. epidermidis*, confirming, again, the above results. In contrast, the diffusion and padding methods are relatively unsuccessful to produce antibacterial fabrics, and they would be less useful for the fixation of GEO into textiles. Interestingly, the antibacterial character remains even after several washing cycles and friction tests (Figure S6). Therefore, a strong correlation between all obtained results indicates the effectiveness of the method used in this study.

## 4. Discussions

The incorporation of EO into textiles, in general, requires chemical treatments, which can reduce the sustainability and ecofriendly properties. In this study GEO were fixed in both cotton and polyester fabrics via environmentally friendly methods such as ultrasound, diffusion, and padding processes, and particularly, ultrasound-assisted absorption of geranium oil allowed the effective fixation of EO into fabric surface in an eco-friendly way.

**Scheme 3:**
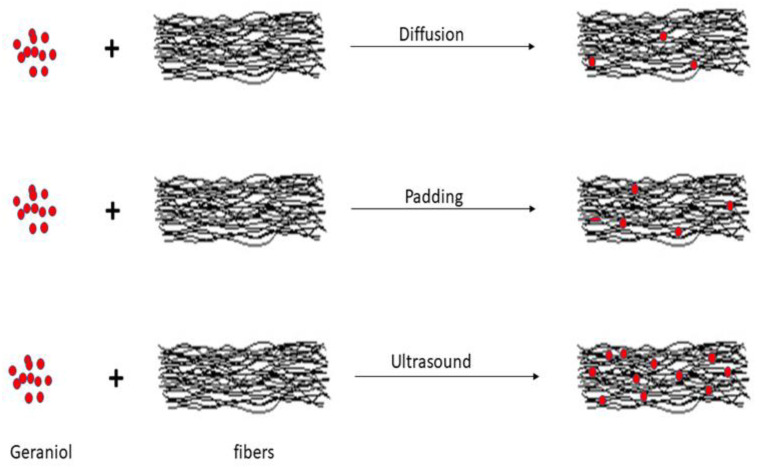
Illustration of the geranium oil absorption into PET and CO fibers via different methods.

The methanol solvent facilitated the penetration of geranium EO on fiber surface and increase its uptake. It is likely that there is an affinity between the functional groups at the surface fiber (like carboxylic and hydroxyl groups) and the molecular bond of EO. Moreover, the increase surface roughness of both CO and PET fiber surface as shown in the SEM image would improve the fixation of EO. Hence, mechanical and physicochemical adhesion between each geranium oil surface and the plasma activated PET or CO fiber surface would be expected to improve the resistance of the immobilized EO removal by washing. The odor intensity of geranium EO immobilized onto CO and P-PET fabrics were analyzed. Absorption of geranium EO has already been reported, hence, the employed method was not ecofriendly and chemicals are used to immobilize the EO [22]. Interestingly, the odor intensity of samples coming from the ultrasound method appeared to be more efficient than the other samples (from the diffusion and padding processes), which could be due to the performance of waves to easily penetrate the EO into fibers. With the three types of treatment method types, ultrasound was recorded during the shortened treatment time, and then the low cost properties were confirmed. It was found that the fabric treated via the diffusion method did not immobilize the geranium EO, which led to the conclusion that ethanol prevents the penetration of EO into fibers and then decreases its concentration. The padding method shows an appreciable result in terms of odor intensity; however, it is not resistant for the washing cycles due to its weak physical interaction of EO at the surface fiber. The odor intensity of EO immobilized on P-PET fabrics was slightly stronger than EO incorporated on CO fabric of a similar treatment method and may be due to the differences in hydrophilic character. The higher antibacterial character of free CO—G and P—PET—G prepared via ultrasound compared to the same materials obtained from diffusion and padding methods might be due to their high amount of geranium EO that is more readily impregnated in water; hence, it increases the contact with bacteria cell, and consequently, improves the inhibition zone. Penetration of geranium EO on the P—PET and CO, using methanol and ultrasound, allows a better penetration of the GEO to help the individual absorption efficiently onto the cotton and polyester fiber surfaces (Scheme 3). It would be expected that increased hydrophilic character of the P—PET—G and CO—G would allow for easy storage of the odor of geranium for more time, thus allowing higher sustainability and reusability.

**Figure 11:**
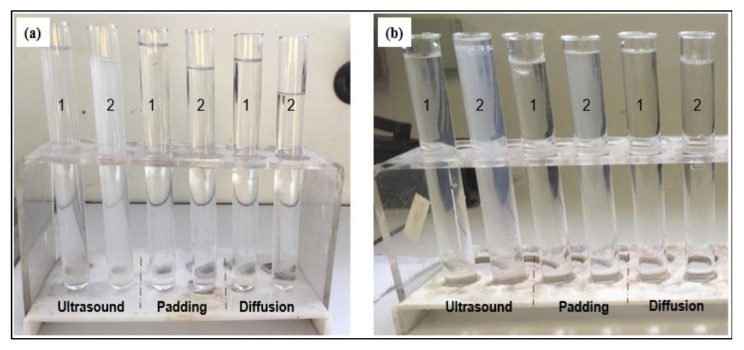
Change on the turbidity of different washing residue for different treatment methods (**a**) after the 1st washing and (**b**) after the third washing cycle, 1: P—PET—G and 2: CO—G.

The interesting results obtained by ultrasound are, presumably, associated to the better mass transfer between solvent and oil in the fabrics. The results are in agreement with the experimental observation, in which the GEO absorbed with high amounts in comparison to the other immobilization methods. The ultrasound approach resulted in higher oil absorption in short preparation time than the padding and diffusion methods. According to Table 3 and the SEM images, possible mechanisms for enhanced oil fixation using ultrasound are as follows: When ultrasound is used in the oil absorption, an ultrasonic wave passes through the solvent creates and collapses the fiber pores. The collapse of the fiber pores produces a violent shock wave and a high-speed jet [38]. The produced shock wave then strikes the surface of the textile fibers, resulting in many pores. Therefore, the methanol as solvent can easily diffuse through the fiber surface, reach the oil droplets embedded in the fabric, dissolve part of the oil droplets, and the oil–solvent solution then diffuse back through the fiber surface. In addition, high turbulence caused by the collapse of the pore favors methanol–geranium oil mixing, thereby increasing the absorption of EO. Thus, the GEO is quickly introduced into the surface fabrics, as supported by the shorter immobilization time. In a word, ultrasound is an effective way to facilitate the penetration of EO into fibrous structures like cotton or polyester fabrics. Additionally, the geranium GEO incorporating fabric demonstrates that it can be recycled at least three times without significantly affecting their performance such as the odor intensity and antibacterial character. Advantageously, the cotton and polyester textiles can be reused after several rub fastness tests. Hence, the washing residue produces a significant confirmation of the above results as explained in Figure 11.

Finally, from the results obtained from both polyester and cotton fabrics, it appears that the ultrasound enhanced the effectiveness of both odor intensity and antibacterial activity in terms of rub fastness, friction, and color strength. This finding not only means that the ultrasound process can be used in an efficient absorption of GEO into fabrics, but also offers the possibility of improving the color strength.

The results showed that ultrasound is more efficient than padding than diffusion, as explained by the high turbidity due to the presence not only of high concentration of geranium oil but also the extent of immobilization and fixation of natural oil components (Figure 11). However, the diffusion method displayed clean residue suggesting that a negligible amount of geranium EO that contains the residue. The results agree with those obtained by SEM, TGA, wettability, olfactory test, and antibacterial analysis.

## 5. Conclusions

Based on this study, geranium oils incorporating textiles are of great importance because of their ecofriendly and antibacterial properties. Three treatments process were evaluated in this work: ultrasound, diffusion, and padding. Results revealed that methanol is an efficient solvent, which facilitates the penetration of EO into the fiber surface. Through the applied method and these findings, ultrasound was selected as an efficient low cost and ecofriendly method for geranium grafting in cotton and polyester fabrics. One important advantage of using the ultrasound method for EO incorporation into textiles and potentially other compounds is to express bioactivity with the lowest energy and consumables. According to the olfactory identification, results suggest that geranium oil immobilized on both cotton and polyester fabrics via ultrasound induced a good odor intensity. In addition, our results support the stability of samples in time that geranium oil persists even after washing and rub fastness cycles. Antibacterial studies concerning suitable methods for the fixation of geranium as EO constituents have confirmed that the ultrasound process is the efficient way to incorporate EO like geranium. Importantly, it was found that the incorporation of EO induced an increase in the color strength for treated fabrics. The results indicate that GEO incorporating polyester and cotton fabrics can cause irreversible damage to the Gram-positive and Gram-negative of microorganism (*S. epidermidis* and *E. coli)*. According the above results, this finding not only means that the ultrasound process can be used in an efficient fixation of essential oil into fabrics, but also offers the possibility to improve the color strength.

## Figures and Tables

**Table 1:** Main parameters of different treatments methods for both PET and CO fabrics.

	Ultrasound	Padding		Diffusion	
Solvent	Methanol	Ethanol	Methanol	Ethanol	Ethanol + Water
Time (min)	30	30	≈10	≈10	240
Temperature (°C)	50	50	30	30	90–130
Consumed water (mL)	0	0	200	200	140
Consumed energy (kJ)	576	15		4140	
Efficiency on GEO fixation	Excellent	Excellent	Average	Average	Low

**Table 2:** Partial Hansen solubility parameters, the total Hildebrand solubility δ_t_ and the thermal stability parameter of geraniol and PET polymer.

	Solubility Parameter (MPa^1/2^)				Thermal Stability (°C)	
	δ_d_	δ_p_	δ_h_	δ_t_	Range of Degradation	Max. Stability
Geraniol	16.9	4.2	7.6	19	80–215	153
PET	18.2	7.3	7.9	21.4	310–450	280

**Table 3:** Evolution of the contact angle for CO, PET and modified counterparts.

Samples	Contact Angle (°) *		
	Ultrasound	Padding	Diffusion
PET	89.95	89.95	89.95
PET—G	73.05	73.51	72.74
CO	73.35	73.35	73.35
CO—G	73.80	73.62	72.97

* The measurements were carried out within 30 s at room temperature.

**Table 4:** Main features of cotton and polyester fabrics and their modified counterparts.

Treatments Method	Samples	Mass Loss (wt. %)		Odour Intensity	Color Strength (K/S)	Color Fastness to Washing Grades **
		78.24 *	416.32 *			
Ultrasound	P—PET	98.77	--	None	0.52	--
	P—PET—G	98.58	89.06	Average-very good	0.91	0.63
	CO	95.12	18.02	None	0.22	--
	CO—G	92.92	19.28	Good-excellent	0.79	0.45
Padding	P—PET	98.77	--	None	0.52	--
	P—PET—G	--	--	Low-average	0.81	0.57
	CO	95.12	18.02	None	0.17	--
	CO—G	--	--	Low-good	0.56	0.40
Diffusion	P—PET	98.77	--	None	0.52	--
	P—PET—G	--	--	Very low-low	0.77	0.62
	CO	95.12	18.02	None	0.22	--
	CO—G	--	--	Very low-low	0.50	0.34

* The temperatures 78 °C and 410 °C were taken as common endsets of the first and second thermal degradation processes only for comparison. ** The K/S values were measured after the third wash fastness cycles.

**Table 5:** Zone of inhibition (mm) of each sample against *E. coli* and *S. epidermidis*.

Methods	Zone of Inhibition (mm)							
	*E. coli*				*S. epidermidis*			
	CO	CO—G	P—PET	P—PET—G	CO	CO—G	P—PET	P—PET—G
Ultrasound	0	3.9	~0	3	~0	3.4	~0	2.7
Padding	0	2	~0	1	~0	1.5	~0	0.5
Diffusion	0	~0	~0	0.1	~0	0.4	~0	0.1

## Data Availability

Data are available on request.

## Supplementary Materials

The following supporting information can be downloaded at: https://www.mdpi.com/article/10.3390/polym15061362/s1, Table S1: Machine parameters of air atmospheric plasma treatment; Table S2: Equivalence of odor intensity according the investigator scores; Figure S1: Olfactory identification ability of geranium oil inserted by different processes on cotton fabrics after the 1st rub fastness test; Figure S2: Comparison of pleasant olfactory character of the treated samples with different treatment methods; Figure S3: Olfactory identification ability of geranium oil inserted by different processes on cotton fabrics after the third rub fastness test. Figure S4: Comparison of pleasant olfactory character of the treated samples with different treatment methods. The results were obtained after the 3rd rub fastness test. Figure.S6: Comparison on the antibacterial activity between ultrasound, diffusion and padding treatment methods. After the 1st and 3rd rub fastness test.
