# Supplementary material for: Assessing Alternative Pre-Treatment Methods to Promote Essential Oil Fixation into Cotton and Polyethylene Terephthalate Fiber: A Comparative Study
Source: Polymers (Basel). 2023 Mar 9;15(6):1362. doi: 10.3390/polym15061362 (PMC10054315; doi:10.3390/polym15061362)
Supplement: Supplementary file 1 [file polymers-15-01362-s001.zip › polymers-2248875-supplementary.pdf]

-Supporting Information-

1. Methods

1.1. Air atmospheric plasma treatment of PET fabrics

.

Table. S1: Machine parameters of air atmospheric plasma treatment

**Parameters**	**Values**
Electrical power	750 w
Frequency	26.0 KHz
Speed	2.0 m/min
Electrode length	0.50 m
Inter-electrode distance	1.50 mm
A treatment power	60 kJ/m^2

1.2. Testing of olfactory function

Table. S2: Equivalence of odour intensity according the investigator scores

**Score**	**Odour Intensity**
0	None
1	Very low
2	Very low
3	Low
4	Low +
5	Average
6	Average +
7	Good
8	Good +

9	Very good
10	Excellent

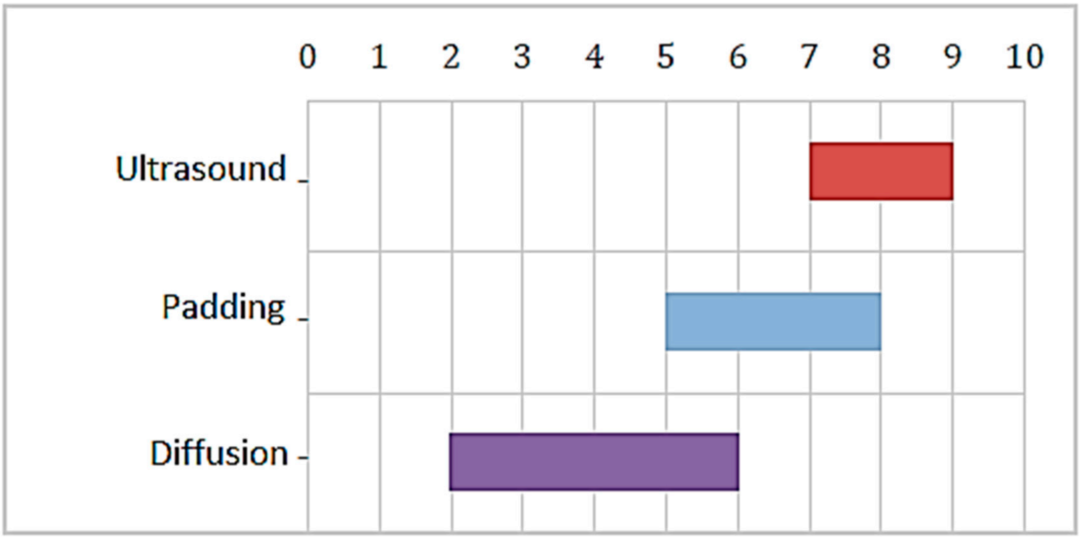

Figure.S1: Olfactory identification ability of geranium oil inserted by different processes on cotton fabrics after the 1st rub fastness test. The figure showed the resistance of geranium oil after the first rub fastness test.

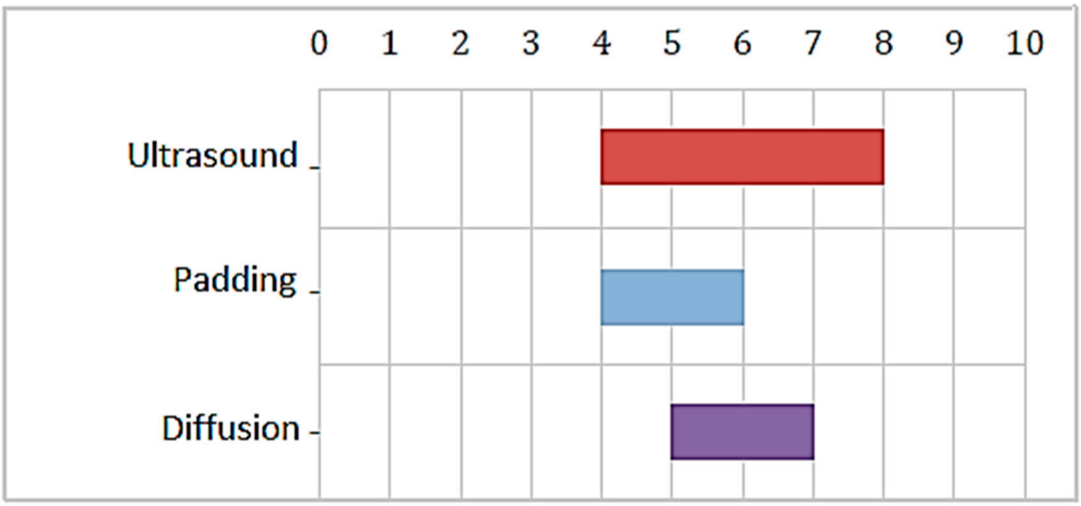

Figure.S2: Comparison of pleasant olfactory character of the treated samples with different treatment methods. The results were obtained after the 1st rub fastness test.

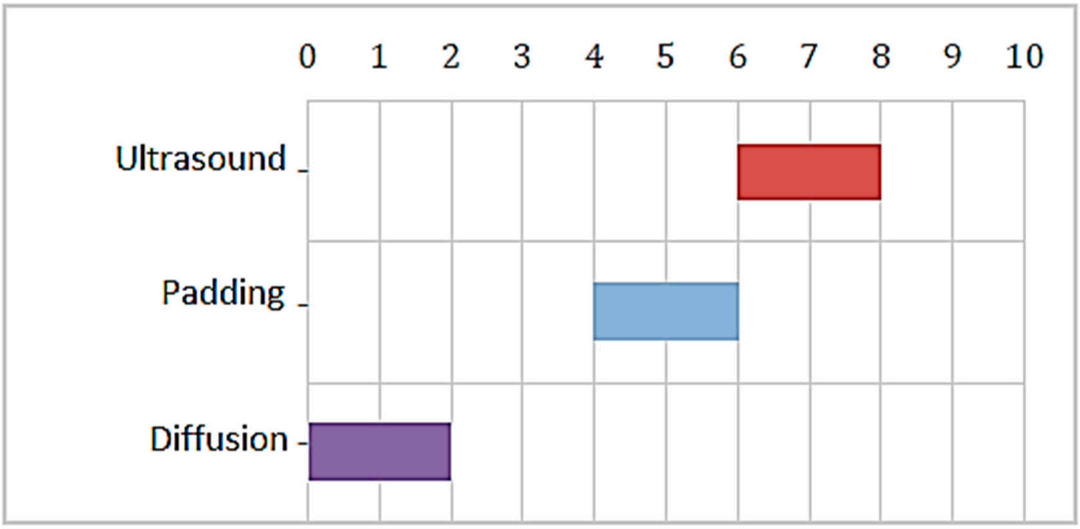

Figure.S3: Olfactory identification ability of geranium oil inserted by different processes on cotton fabrics after the 3rd rub fastness test. The figure showed the resistance of geranium oil after the third rub fastness test.

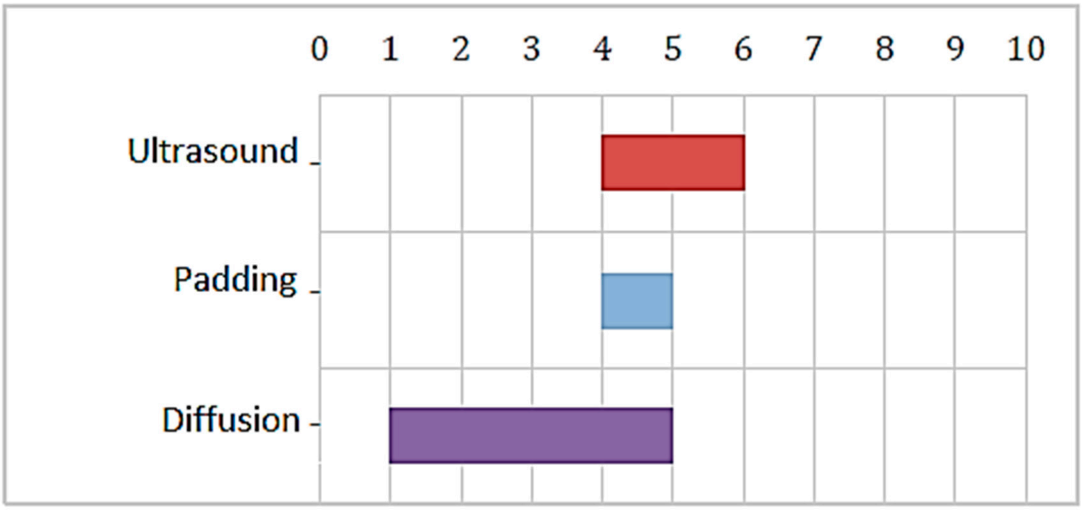

Figure.S4: Comparison of pleasant olfactory character of the treated samples with different treatment methods. The results were obtained after the 3rd rub fastness test.

1. Antibacterial measurements

<table>
<tr><th>Ultrasound</th><th>Padding</th><th>Diffusion</th></tr>
<tr><td colspan="3">After the 1st rub fastness</td></tr>
<tr><td>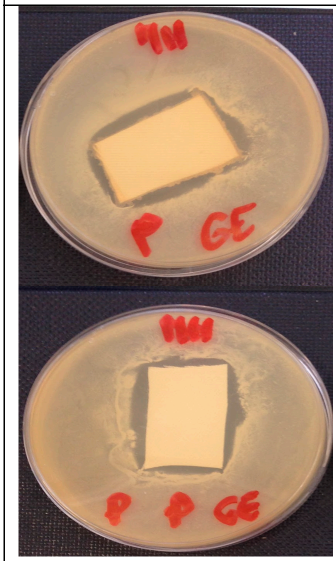</td><td>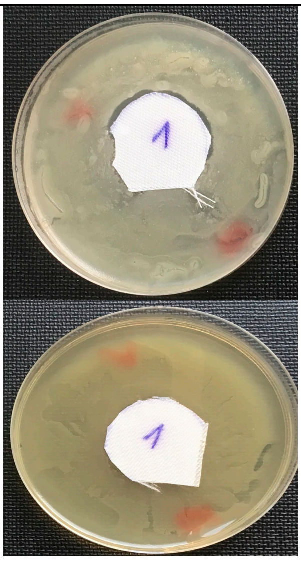</td><td>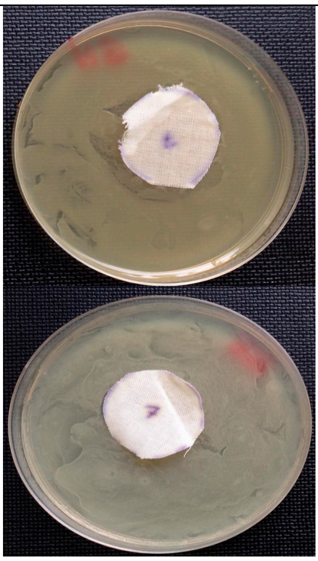</td></tr>
<tr><td colspan="3">After the 3rd rub fastness</td></tr>
<tr><td>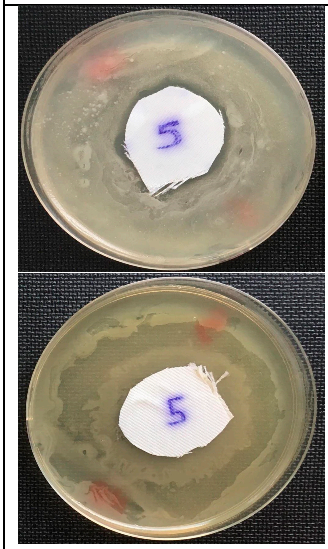</td><td>No antibacterial characters</td><td>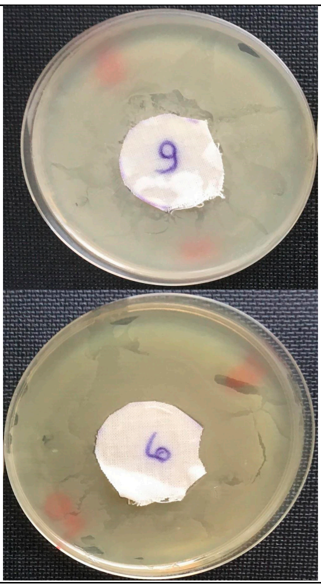</td></tr>
</table>

Figure.S6: Comparison on the antibacterial activity between ultrasound, diffusion and padding treatment methods. After the 1st and 3rd rub fastness test.
